# Supplementary material for: The AKT isoforms 1 and 2 drive B cell fate decisions during the germinal center response
Source: Life Sci Alliance. 2019 Nov 25;2(6):e201900506. doi: 10.26508/lsa.201900506 (PMC6878223; doi:10.26508/lsa.201900506)
Supplement: Supplementary file 1 [file LSA-2019-00506_Supplemental_Data_1.docx]

Supplementary Material

Materials and Methods

Mice

*Akt1*^f/f^ (Cho et al, 2001), *Akt2*^-/-^ (Dummler et al, 2006), and *Akt3*^-/-^ (Tschopp et al, 2005) were kindly provided by Dr. Morris J. Birnbaum (University of Pennsylvania, USA). *Bcl2* transgenic mice (*Eμ-bcl-2-22*) (#002319) were purchased from The Jackson Laboratory. *CD19*^Cre^ (Rickert et al, 1997) and *Cγ1*^Cre^ (Casola et al, 2006) mice have been previously described. All studies and procedures were conducted with mice 8-16 wks of age and approved by the Animal Review Committee in SBP.

Flow cytometry analysis

Mouse splenic B cells were purified by negative selection using CD43-specific beads, followed by separation on MACS columns (Miltenyi Biotec). B cell purity was >98% as measured by FACS analysis using anti-B220 antibody (Thermo Fisher Scientific).

For surface staining, cells were blocked with anti-CD16/32 (clone 2.4G2; BD Biosciences) and stained with the indicated combination of fluorochrome conjugated antibodies for 30 min on ice. The following antibodies were obtained from Thermo Fisher Scientific: anti-B220 (clone: RA3-6B2), -IgM (clone: II/41), -CD69 (H1.2F3), -CD86 (clone: PO3.1), -GL7 (clone: GL-7 (GL7)), -MHC class II (clone: M5/114.15.2), anti-AA4.1 (clone: AA4.1). Anti-CD43 (clone: S7), -CD80 (clone: 16-10A1), -CD184 (Clone: 2B11/CXCR4), and -FAS (clone: Jo2) were purchased from BD Biosciences. Unconjugated anti-GLUT1 (clone: SA0377) was purchased from Invitrogen.

For intracellular staining, cells were fixed and permeabilized using BD Cytofix/Cytoperm Buffer (BD Biosciences) for 15 min at room temperature. Cells were subsequently washed with 1 x BD Perm/Wash buffer (BD Biosciences) prior to staining with fluorochrome conjugated antibodies. The following antibodies were obtained from BD Biosciences: anti-p-BLNK (Y84) (clone: J117-1278), [-PLC-γ2 (pY759)](http://www.bdbiosciences.com/us/applications/research/intracellular-flow/intracellular-antibodies-and-isotype-controls/anti-human-antibodies/pe-mouse-anti-plc-2-py759-k86-68937/p/558490) (clone: K86-689.37). Anti-p-Erk1/2 (T202/Y204) (Clone: D13.14.4E) were purchased from Cell Signaling Technology.

All cells were acquired on a FACSCanto flow cytometer using the FACSDiva software (BD Biosciences) and data were analyzed using FlowJo software (Tree Star).

Immunizations and ELISA

To study affinity maturation, mice were i.p. immunized with 50 μg alum precipitated NP25-CGG (Biosearch Technologies). Serum was collected on day 7, day 14, and day 21 after immunization. Antibody titers were determined by ELISA. NP4-BSA and NP23-BSA coated plates were used to detect high affinity antibodies and total titers, respectively.

Western blot analysis

Whole cell lysates were prepared by direct lysing with RIPA buffer plus complete protease inhibitor mixture (Roche). Protein concentration of whole cell lysates was quantitated using the BCA protein assay kit (Pierce). Samples were boiled in NuPAGE™ LDS Sample Buffer (Invitrogen) supplemented with β-mercaptoethanol and then were separated by 4−12% polyacrylamide Bis-Tris gel (Bio-Rad) and transferred onto polyvinylidene difluoride membrane (EMD Millipore). The membrane was probed for the indicated proteins. The following antibodies were purchased from Cell Signaling Technology: anti-AKT1 (clone: C73H10), -AKT2 (clone: D6G4), -AKT3 (clone: E1Z3W), -total AKT (Cat#: 9272), -β-actin (clone: 13E5), -cyclin D3 (Clone: DCS22), -mTor (Cat#: 2983) -Erk1/2 (Cat#: 9102), -p-Erk1/2 (T202/Y204) (Clone: D13.14.4E), -FOXO1 (Clone: C29H4), -p-FOXO1 (S256) (Cat#: #9461), -p-FOXO1 (T24A) (Cat#: 9272), -p-S6 (S240/244) (Clone: D68F8). Anti-Mcl-1 (Cat#: 600-401-394S) were purchased from Rockland Immunochemicals. Primary antibodies were then detected with HRP-labeled donkey anti-rabbit antibodies (Jackson Immuno-Research). HRP antibody target proteins were detected by incubating with Supersignal West Pico chemiluminescent substrate (Thermo Fisher Scientific) and exposing the blot to x-ray film. Protein bands were quantitated with Image J software (NIH, Bethseda, MD).

Glucose uptake assay

Glucose uptake was measured with 2-NBDG (Invitrogen). Briefly, freshly isolated B cells were unstimulated or stimulated as indicated for 24 hours. The cells then were washed with PBS, and resuspended in glucose free RPMI 1640 medium (Gibco) in the presence of 100 μM 2-NBDG and cultured for 15 minutes at 37°C prior to flow cytometry analysis.

Statistical analysis

Results are expressed as mean ± SEM and were determined with one-way ANOVA with the post-hoc Tukey’s multiple comparisons test and the student’s t test for unpaired samples. Statistical significance is indicated by asterisks (*, P < 0.05; **, P < 0.01; and ***, P < 0.001), with P values >0.05 considered non significant (ns).

Supplementary References

Casola S, Cattoretti G, Uyttersprot N, Koralov SB, Seagal J, Hao Z, Waisman A, Egert A, Ghitza D, Rajewsky K (2006) Tracking germinal center B cells expressing germ-line immunoglobulin gamma1 transcripts by conditional gene targeting. *Proc Natl Acad Sci U S A* 103: 7396-7401. doi: 10.1073/pnas.0602353103.

Cho H, Thorvaldsen JL, Chu Q, Feng F, Birnbaum MJ (2001) Akt1/PKBalpha is required for normal growth but dispensable for maintenance of glucose homeostasis in mice. *J Biol Chem* 276: 38349-38352. doi: 10.1074/jbc.C100462200.

Dummler B, Tschopp O, Hynx D, Yang ZZ, Dirnhofer S, Hemmings BA (2006) Life with a single isoform of Akt: mice lacking Akt2 and Akt3 are viable but display impaired glucose homeostasis and growth deficiencies. *Mol Cell Biol* 26: 8042-8051. doi: 10.1128/MCB.00722-06.

Rickert RC, Roes J, Rajewsky K (1997) B lymphocyte-specific, Cre-mediated mutagenesis in mice. *Nucleic Acids Res* 25: 1317-1318. doi: 10.1093/nar/25.6.1317.

Tschopp O, Yang ZZ, Brodbeck D, Dummler BA, Hemmings-Mieszczak M, Watanabe T, Michaelis T, Frahm J, Hemmings BA (2005) Essential role of protein kinase B gamma (PKB gamma/Akt3) in postnatal brain development but not in glucose homeostasis. *Development* 132: 2943-2954. doi: 10.1242/dev.01864.
